# Anatomical Bill

**Published:** 1884-07

**Authors:** 


### ANATOMICAL BILL.

The bill to amend the present law regarding the disposition of unclaimed dead, framed by the Demonstrators' Association of Chicago, lies before us. A letter from the Secretary of the Association, Dr. A. B. Strong, 533 West Monroe street, Chicago, with a copy of the bill, has been sent to all physicians in the State, soliciting their aid, and requesting them to interview in person the members of the next Legislature, and secure as many friends for the bill as possible. Thus far, replies have been received from over 300 physicians, promising their active support.

If the members of the profession in this State will interest themselves in this matter there can be no doubt of the adoption of the amendment next winter. The failure in the original bill, which placed it in the power of a few local politicians to defeat the will of the people and the evident intention of the General Assembly, should be remedied at once. The following are a few extracts from the Secretary's stirring letter:

"The existing law has been satisfactory in its working until the last year; fulfilling in every particular the intention of the law; that is, preventing the desecration of cemeteries and benefitting alike medical students, physicians, and the general public by furnishing the colleges a plentiful supply of material from the unclaimed pauper dead. The existing law is weak and deficient in the following particular: It is not mandatory—says *may* instead of *shall* when designating what bodies may be delivered to medical colleges; thus giving designing persons an opportunity to interfere with the intention of the law. * * * The only remedy is additional legislation. The accompanying draft of a bill amends the present law by, first, making it mandatory, and thus prevents any interference with its proper intentions, by a political clique, or other meddling persons. Second, giving preceptors as well as teachers in medical colleges the right to claim material for medical and surgical uses. Now, Doctor, knowing, as you do, the necessity for dissection and experimental operation, may we not rely on you to give what personal aid is in your power to advance the cause of medical education in and out of college, by making it a matter of personal interest; to call upon the members of your district and urge them to support the measure for the following reasons among others:

"First: The dissection of human bodies is absolutely necessary for the proper education of the physician.

"Second: Such being the case, the colleges must and will have human bodies.

"Third: There are bodies enough of those unclaimed and unknown persons who die in the institutions supported by public tax, to meet the demands of the colleges.

"Fourth: Consequently, the law is a protection to private cemeteries; for since the passage of the present law until this winter, public morals and decency have not been once outraged by the fiendish acts of the resurrectionist. Will you please advise me of the names and addresses of legislators whom you may see, with their opinions of the bill, that we may have some idea of the number of its friends in the next Legislature."

Every physician in this State, every one interested in the welfare of our profession and a higher and better medical education should give this bill his hearty support. Show the general public and politicians that doctors, though they differ at times, are a unit in what concerns the welfare and ultimate good of the profession.

We hope that every one of our readers residing in the State of Illinois will coöperate with Dr. Strong in his efforts to put this amendment on our statute books.

---

We call the attention of our readers to the list of Boylston prize questions, for 1885–86, among the advertisements. It is to be hoped that some of our readers in the West will gain one of these prizes, so rich in professional honors and pecuniary rewards.

